# Analysis of Wireless Sensor Network Topology and Estimation of Optimal Network Deployment by Deterministic Radio Channel Characterization

**DOI:** 10.3390/s150203766

**Published:** 2015-02-05

**Authors:** Erik Aguirre, Peio Lopez-Iturri, Leire Azpilicueta, José Javier Astrain, Jesús Villadangos, Francisco Falcone

**Affiliations:** 1 Electrical and Electronic Engineering Department , Universidad Pública de Navarra, Pamplona 31006, Spain; E-Mails: aguirrerik@gmail.com (E.A.); peio.lopez@unavarra.es (P.L.-I.); leyre.azpilicueta@unavarra.es (L.A.); 2 Computer and Mathematics Engineering Department, Universidad Pública de Navarra, Pamplona 31006, Spain; E-Mails: josej.astrain@unavarra.es (J.J.A.); jesusv@unavarra.es (J.V.); 3 Smart Cities Institute, Universidad Pública de Navarra, Pamplona 31006, Spain

**Keywords:** wireless sensor networks, 3D ray launching, IEEE 802.15.4, WSN system, level simulation

## Abstract

One of the main challenges in the implementation and design of context-aware scenarios is the adequate deployment strategy for Wireless Sensor Networks (WSNs), mainly due to the strong dependence of the radiofrequency physical layer with the surrounding media, which can lead to non-optimal network designs. In this work, radioplanning analysis for WSN deployment is proposed by employing a deterministic 3D ray launching technique in order to provide insight into complex wireless channel behavior in context-aware indoor scenarios. The proposed radioplanning procedure is validated with a testbed implemented with a Mobile *Ad Hoc* Network WSN following a chain configuration, enabling the analysis and assessment of a rich variety of parameters, such as received signal level, signal quality and estimation of power consumption. The adoption of deterministic radio channel techniques allows the design and further deployment of WSNs in heterogeneous wireless scenarios with optimized behavior in terms of coverage, capacity, quality of service and energy consumption.

## 1. Introduction

The complexity of indoor scenario characterization concerns signal propagation, but also interferences caused by the coexistence of different communication systems working in the same frequency band or by the congestion due to abundant deployment of wireless devices. A seasoned designer of wireless sensor networks (WSN) usually quite easily identifies which are the most suitable sites to locate the nodes of the network, but a subsequent experimental validation is required in order to ensure that the decision is correct and that the network operates in accordance with the requirements set by the customer. For such reason, simulation, modeling and analysis of WSNs are required.

As it is well known, WSNs need to minimize the energy consumption of their nodes to ensure the longevity of the networks, and maximize service performance. For such a reason, an inefficient network design may cause high energy consumption due to an inappropriate selection of the topology of the network and an unsuitable location of the nodes. The different existing proposals for routing in WSNs focus on the use of clusters, chains or hybrid solutions. LEACH [1] is the reference proposal on cluster-based WSNs, a cluster-head collects data from all other sensors in the cluster, aggregates the data, and then transmits the whole information to a base station, also called sink. LEACH rotates the cluster-head duty in order to evenly distribute the energy consumption. PEGASIS [2,3] forms, by means of a greedy algorithm, a chain covering all the nodes in the network, ensuring that each node only communicates with its neighbors. The transmission of the aggregated information is performed by a node which is randomly selected each round to uniformly distribute the energy consumption. Cluster- based topologies ensure fault tolerance, but they do so at the expense of a higher energy consumption due to collisions and retransmissions [4]. Chain-based topologies follow a space filling curve that ensures a minimal consumption of battery nodes due to transmission and reception [5]. In this case nodes minimize the number of messages exchanged and then battery consumption decreases in both transmission and reception modes. Other authors propose hybrid methods trying to maximize the advantages of both clusters as chains (fault tolerance, minimal energy consumption), while minimizing their disadvantages (latency, cost of chain regeneration, high amount of messages exchanged) as [6] do achieving the maximum reliability in a multi-hop network by finding the best place for the cluster-head and the proper shape/size of the clusters without requiring any error controlling minimizing computation and communication overheads. Mahajan *et al.* [7] proposed the formation of sensor nodes chains in each cluster rotating the cluster-head locally inside the cluster without re-clustering. Goyeneche *et al.* [8] proposed a distributed algorithm in order to avoid centralized information about energy consumption when agreeing the cluster-head. Those proposals [6–9] seek to improve the energy consumption and to increase the lifetime of the WSN compared to other classical hierarchical routing schemes such as LEACH and PEGASIS, but significantly increase the complexity of the algorithms.

Indoor scenarios can be covered using chain-based routing protocols, where an adequate radio planning ensures that the chain should be rarely redone. This is the case addressed on this paper, where the whole process of designing and deploying a chain-based WSN by means of in-house 3D ray launching simulation is illustrated. The paper describes the results obtained by simulation and compares them with those obtained by real measurements, proving that an in-house 3D ray launching simulation is an efficient method for reducing the time and effort required to design and develop WSNs on such scenarios. The influence of network topology is explicitly considered in order to provide the optimal location of wireless transceivers, in terms of received power levels and interference margins, leading to the final deployment proposed by the network designer and to the radioplanning strategy. One of the main challenges in the analysis of future Internet of Things and Context-Aware Scenarios is to consider the full complexity in terms of overall dimensions and the large amount of elements, in which a potentially vast amount of sensors and transceivers can be located. Different methods can be applied, such as empirical estimations or full wave electromagnetic simulation. The first are strongly site-specific and require intensive measurement campaigns, which usually leads to large average errors in the estimation. The latter are very precise but exhibit very large computational demands, which for large scenarios limit their use in practical terms. Another approach is to use deterministic based algorithms, based on Geometric Optics and Uniform Theory of Diffraction, in which rays launched from the source resemble for a discretized electromagnetic wave-front. The implementation of this method is usually bi-dimensional (in horizontal, vertical or simultaneously both planes), although three dimensional implementations have also been done. In this work, an in house implemented 3D Ray Launching code is employed, which has been specifically adapted to the analysis of scenarios in which wireless sensors and transceivers can be allocated in multiple positions. This approach provides estimations of multiple quality assurance metrics (such as coverage levels, time dependent parameters such as power delay profiles and more elaborate estimators, such as current consumption), in an alternative manner as the common empirical/statistical solutions commonly reported.

The rest of the paper is organized as follows: Section 2 is devoted to introduce the indoor scenario as well as the in-house 3D Ray Launching simulation; Section 3 describes the experimental methodology, while Section 4 validates the results obtained by simulation and those obtained by diverse measurement procedures. Finally, conclusions and references end the paper.

## 2. Indoor Scenario Simulation and Characterization

In order to estimate the feasibility of deploying WSNs inside indoor complex environments, a radio coverage map becomes essential. Various indoor models have been developed in the past based on semi-empirical approaches [10–12]. The advantage of these methods is that they require low computational cost but they have limited accuracy in comparison with high precision full wave techniques, which also exhibit very high computational cost [13–19]. A commitment between accuracy and computational time is acquired with deterministic methods, which are based on Geometrical Optics [20]. The Ray Tracing approaches combined with uniform theory of diffraction (UTD) is most commonly employed to radio coverage estimation [21–24].

**Figure 1.**
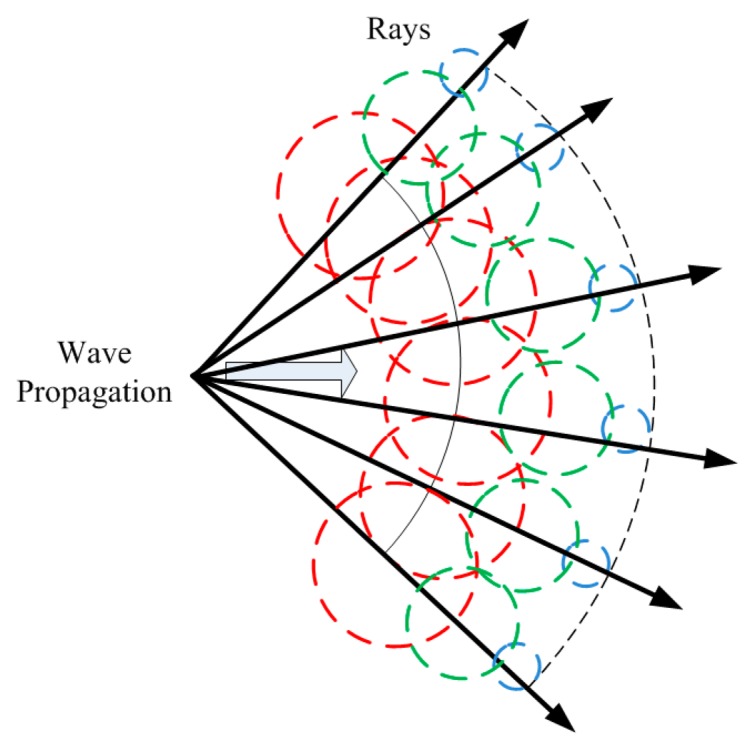
Wave front propagation with rays associated with single wave front points.

As illustrated in Figure 1, the ray launching (RL) technique is based on recognizing a single point of the radiated wave with a ray that propagates along the space following a combination of optic and electromagnetic theories. RL techniques can be used in environments where the frequency of interest is much smaller than the dimensions of the surroundings.

A 3D in-house developed RL algorithm has been used for the characterization of wireless propagation in the ground floor of a department of the Public University of Navarre (UPNA, Pamplona, Spain). The algorithm has been explained in detail in [25] and it has been validated in the literature for different applications, like the analysis of wireless propagation in closed environments [26–29], interference analysis [30] or electromagnetic dosimetry evaluation [31]. It is based on Geometrical Optics (GO) and Geometrical Theory of Diffraction (GTD). GO approaches consider only direct, reflected and refracted rays, bringing about abrupt areas, which correspond with the boundaries of the regions where these rays exist. Due to this constraint, the diffracted rays are introduced with the GTD and its uniform extension, the Uniform GTD (UTD).

**Figure 2.**
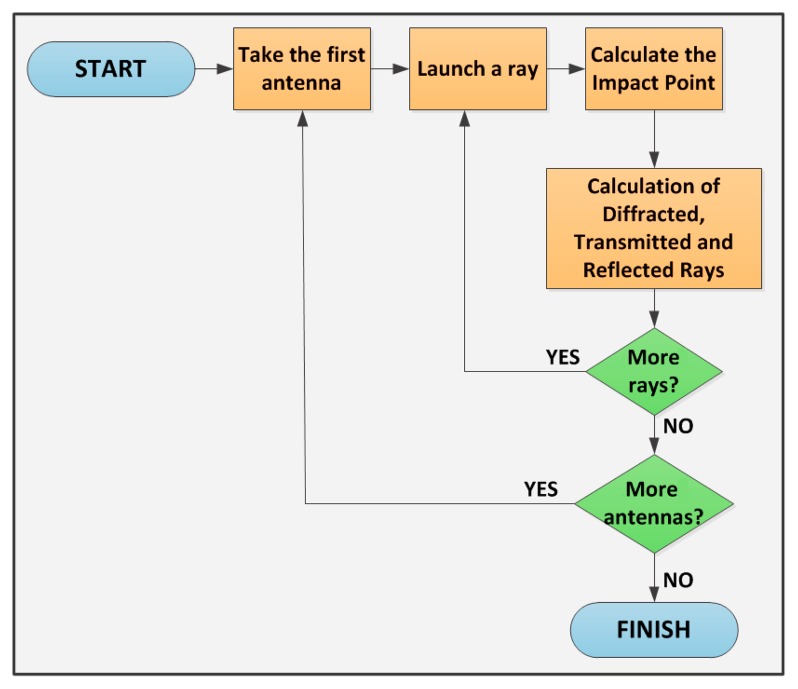
Functional diagram of the 3D ray launching algorithm implemented at Public University of Navarre.

The whole scenario under analysis is divided into a uniform hexahedral mesh with cuboids of a given dimension. Rays at a predetermined power level are launched in a predefined solid angle from a specific transmitter location and the resulting power levels along the path are calculated and stored in the respective cuboids. The rays will be reflected, transmitted and diffracted by the structure of the walls, the furniture of the offices, the windows, the seats, *etc.* The material properties for all the elements within the scenario are considered, given the dielectric constant and permittivity at the frequency range of operation of the system under analysis. Figure 2 summarizes the main points of the basic principle of the developed RL algorithm.

Each ray propagates in the space as a single optic ray. The calculation of the electric field *E* created by an antenna with *P_rad_* as radiated power, with a directivity *D_t_*(θ*_t_*, Φ*_t_*) and polarization ratio (*X*^⊥^, *X*^∥^) at a distance *d* in the free space is calculated by [32]:

(1) $$E_{i}^{⊥}=\sqrt{\frac{P_{\text{rad}}D_{t}(\theta_{t},\emptyset_{t})\eta_{0}}{2\text{Π}}}\frac{e^{-j\beta_{0}r}}{r}{\text{X}}^{⊥}L^{⊥}$$

(2) $$E_{i}^{∥}=\sqrt{\frac{P_{\text{rad}}D_{t}(\theta_{t},\emptyset_{t})\eta_{0}}{2\text{Π}}}\frac{e^{-j\beta_{0}r}}{r}{\text{X}}^{∥}L^{∥}$$

where $\beta_{0}=2\pi f_{c}\sqrt{{ɛ}_{0}\mu_{0}}$, ε_0_ = 8.854 × 10^−12^, μ_0_ = 4π × 10^−7^ and η_0_ = 120π. *L*^⊥∥^ are the path loss coefficients for each polarization. A reflected ray and a transmitted ray are created with new angles provided by Snell’s law [33] when this ray finds an object in its path. The new angles (θ*_r_*, Φ*_r_*) of the reflected wave and (θ*_t_*, Φ*_t_*) of the transmitted wave are calculated once the parameters of transmission *T* and reflection *R* are calculated, and the angle of incidence ψ_i_ and ψ*_t_*.

**Figure 3.**
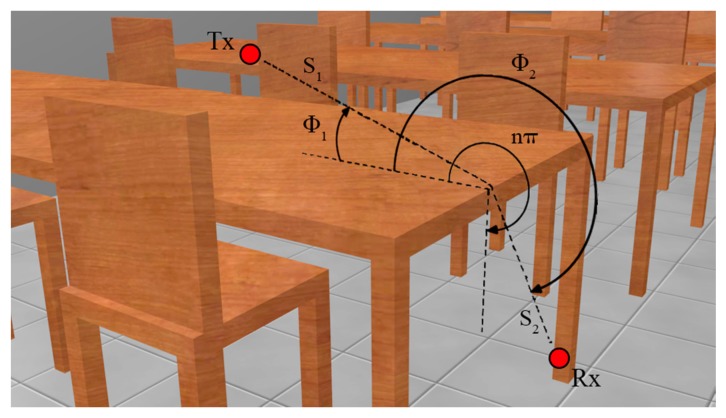
Geometry for wedge diffraction coefficients.

The diffracted field is calculated by [34]:

(3) $$E_{\text{UTD}}=e_{0}\frac{e^{-jks_{1}}}{s_{1}}D^{⊥∥}\sqrt{\frac{s_{1}}{s_{2}(s_{1}+s_{2})}}e^{-jks_{2}}$$

where *s*_1_, *s*_2_ are the distances from the source to the edge and from the edge to the receiver point, respectively. *D*^⊥∥^ are the diffraction coefficients given by [34–36] as:

(4) $$D^{∥⊥}=\frac{-e^{\left(-j\pi /4\right)}}{2n\sqrt{2\pi k}}\left\{\begin{array}{c} \cot\left(\frac{\pi +\left(\Phi_{2}-\Phi_{1}\right)}{2n}\right)F\left(kLa^{+}\left(\Phi_{2}-\Phi_{1}\right)\right)\\ +\cot\left(\frac{\pi -\left(\Phi_{2}-\Phi_{1}\right)}{2n}\right)F\left(kLa^{-}\left(\Phi_{2}-\Phi_{1}\right)\right)\\ +R_{0}^{∥⊥}\cot\left(\frac{\pi -\left(\Phi_{2}+\Phi_{1}\right)}{2n}\right)F\left(kLa^{-}\left(\Phi_{2}+\Phi_{1}\right)\right)\\ +R_{n}^{∥⊥}\cot\left(\frac{\pi +\left(\Phi_{2}+\Phi_{1}\right)}{2n}\right)F\left(kLa^{+}(\Phi_{2}+\Phi_{1})\right) \end{array}\right\}$$

where *n*π is the wedge angle, *F*, *L* and *a* ± are defined in [34], *R*_0,*n*_ are the reflection coefficients for the appropriate polarization for the 0 face or n face, respectively. The Φ_2_ and Φ_1_ angles in Equation (4) would refer to the angles in Figure 3.

With the parameters stored in each hexahedron of the considered scenario, the received power can be calculated at each point taking into account the losses of propagation through a medium (ε, μ, σ) at a distance *d*, with the attenuation constant α (Np/m), and the phase constant β (rad/m). Based on this theory, the main contribution of the ray-launching technique is that it provides the impulse response of the channel *h* (*t*, *fc*, Δ*f*, *r*) for each transmitter, at a given carrier frequency, *fc*, at a given bandwidth (*fc* ± Δ*f*), where the materials have a similar response and at a given position, *r*. A stationary channel can be wholly characterized with this information. Three dimensional maps of power levels can be obtained, as well as delay spread maps, power delay profiles and Signal to Noise ratio levels.

**Figure 4.**
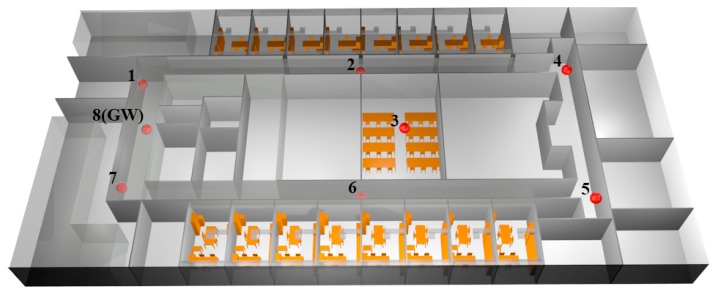
Schematic representation of the simulated scenario with the location of the antennas (represented by red points).

The entire floor of the Public University of Navarre department building has been considered for the simulations, taking into account all the furniture and infrastructures as chairs, tables or air ducts, as depicted in Figure 4. The total length of the scenario is 55.3 m × 27.85 m × 3.8 m and it has been divided into 1 m size cuboids. Eight antennas have been distributed throughout the scenario, considering the real antennas properties and the locations used in the measurement campaign. The configured parameters for the simulations are given in Table 1.

**Figure 5.**
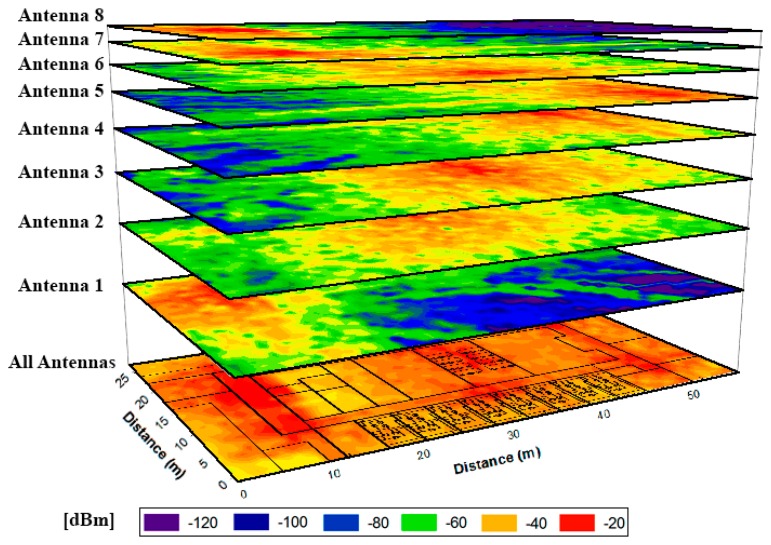
RF power contribution of the simulated antennas for the plane at height = 1 m, individually as well as the total power distribution (bottom plane).

The coverage given by a single antenna is not capable of providing service for the complete scenario, but an adequate RF power distribution can be obtained deploying properly the eight elements of the proposed network. This behavior is shown in Figure 5, where the contribution of each antenna separately and the jointly contribution are depicted.

**Figure 6.**
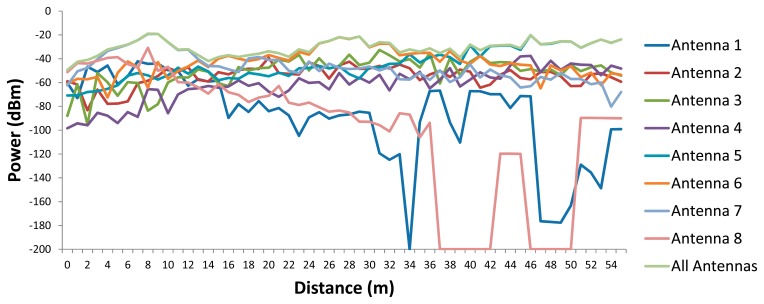
Estimated received power *vs.* linear distance for the antennas transmitting separately and together.

For a more in depth analysis of the results shown in Figure 5, in Figure 6 the estimated received power *vs.* linear distance for the aisle where antennas 5, 6 and 7 are placed (see Figure 4) is shown.

The results show the typical signal strength variations due to the multipath propagation, as well as how a unique antenna cannot provide service for the whole scenario (the sensibility of the used wireless devices is −92 dBm), making necessary the deployment of more wireless nodes.

**Figure 7.**
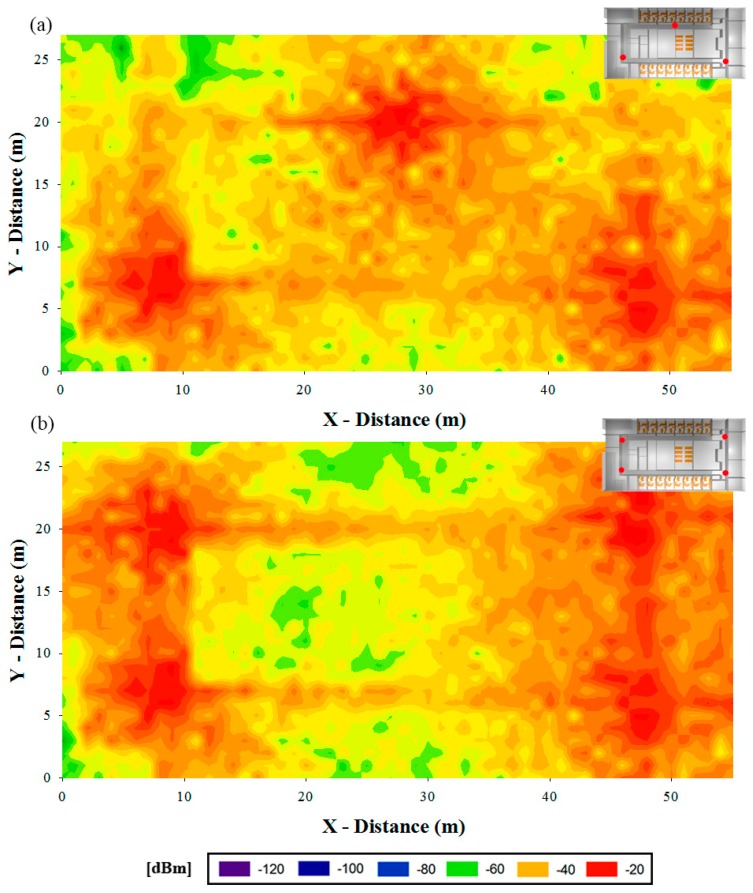
Preliminary distribution of antennas where three (**a**) and four (**b**) antennas have been considered.

The result presented in Figure 5 is the consequence of testing different antenna emplacements and configurations and choosing the most suitable in terms of coverage and power consumption. Therefore, with the aim of prove the necessity of a correct characterization before the implementation of the system, two preliminary cases are depicted in Figure 7. This process is inherent to the radioplanning strategy in order to provide the final network configuration, proposed in last term by the network designer.

The theoretical study of the scenario allows the consideration of multiple configurations avoiding the tedious work of test different implementations of the system until the most optimum configurations is obtained. In the two cases presented in Figure 7 it is visible a coverage result that is unsuitable since the complete scenario does not exhibit an acceptable received power value level.

**Figure 8.**
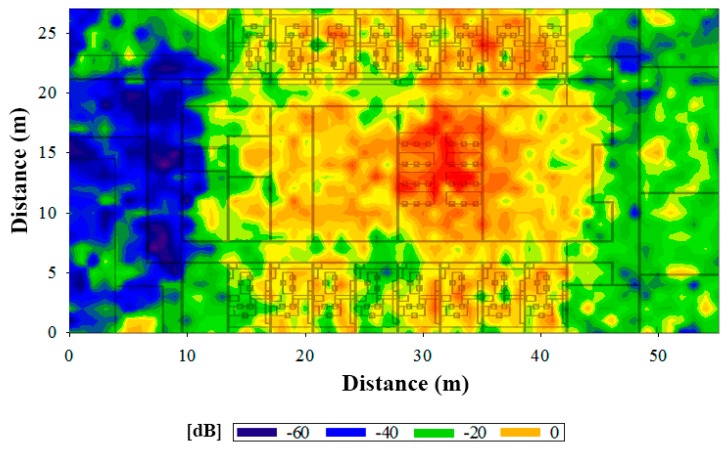
SNR map with the interference caused by all the antennas over the third antenna acting as valid transmitter.

One of the most limiting factors in WSN performance is given by receiver sensitivity, determined basically by hardware limitations (such as noise factor) and employed modulation and coding schemes, which in turn determine maximum tolerable interference levels. The possible interference produced between transmitters must be considered in order to reduce packet loss and optimize the communication. Based on the results extracted from mathematical simulations, an efficient sensor mesh can be constructed, therefore in Figure 8 a horizontal plane is depicted where the interference caused by all the antennas over third antenna, acting as potential valid transmitter is shown.

**Figure 9.**
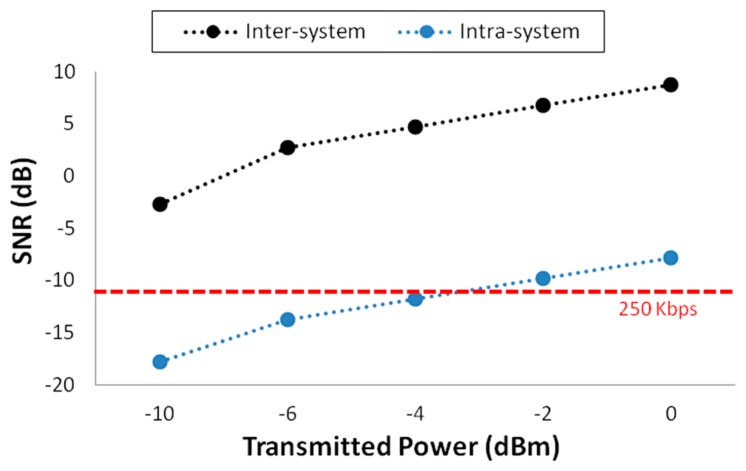
Estimated inter-system and intra-system SNR values for antenna 3.

Related with this, in Figure 9 estimations of SNR level at the point where antenna 4 is placed are shown. This is the case of antenna 3 acting as the transmitter which communicates with node 4 in the chain routing protocol, considering the rest of devices intra-system interference (transmitting at maximum power level, 0 dBm). The same SNR estimations have been calculated for inter-system interference, considering −55 dBm of noise level, an average value extracted from a spectrogram measured in the scenario before the deployment of the ZigBee network. With the aim of determining if the communication between the two wireless devices is possible, five SNR values at position 4 have been calculated, one for each of the five transmitted power levels allowed by the devices used for measurements. The minimum required SNR value for a correct transmission between these two devices has been calculated using the following well known formula:

(5) $$\left[C=BW\times {\text{log}}_{2}\;\left(1+\frac{S}{N}\right)\right]$$

where *C* is the channel capacity (250 Kbps, maximum value for ZigBee devices) and BW is the Band-Width of the channel (3 MHz).

As can be seen, there is not an inter-system interference problem in the presented scenario for the communication between devices 3 and 4. On the other hand, a failed communication could happen with intra-system interference in some case, although it is nearly the worst case possible: transmitting at maximum data rate and the interfering devices transmitting at highest power level (0 dBm). Under these conditions, the communication is viable only when the transmission power level is 0 dBm or −2 dBm. Therefore, our simulation tool can help estimating the SNR value at each point of the scenario, allowing the designer making the correct decision in order to deploy the devices and configuring them in an energy-efficient way.

**Figure 10.**
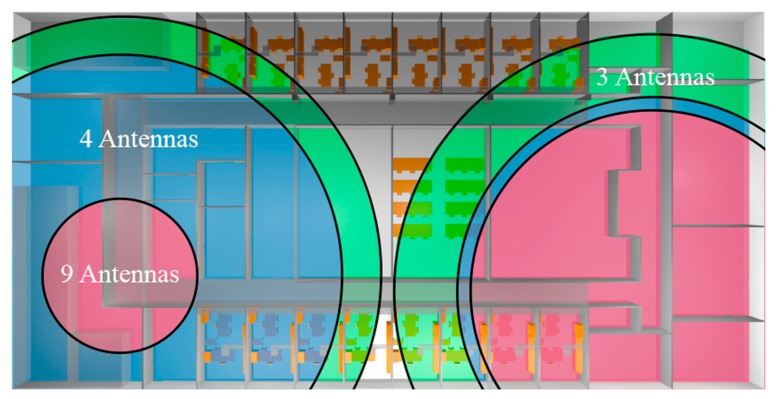
Approach to the maximum emitting distance for three configurations of antennas according to −10dB SNR threshold considering that 5th (**Left**) or 7th (**Right**) antenna is emitting.

In terms of SNR, the selected configuration could not be as suitable as some preliminary tested scenarios. Continuing with the examples shown in Figure 4 where three and four antennas are considered, the SNR levels are higher since more antennas suppose more intra-system noise. In Figure 10 the approximation of the range that can be covered until the −10dB SNR threshold is reached with the three different configurations is shown. In this figure it is considered that antenna 5 or 7 is emitting and that the rest of antennas are noise.

As it is visible when the three antenna configuration is employed, the area where SNR is acceptable is larger than in other cases. Moreover, since more antennas are placed next to the 7th antenna in the definitive configuration, the SNR area is smaller than when antenna #5 is transmitting. Note that in the real system the power received from at least one of the transmitters must be considered as valid information and not as noise/interference. In fact, according with the represented circles in some cases the noise level is too high to allow a correct data transmission. These variations in noise floor levels can be given by variations in the density of mobile transceivers (*i.e.*, mobile users in the vicinity of the transmitter antennas within the scenario), which must be determined empirically. Nevertheless, as it is visible in Figure 9 and in the experimental results presented later on, the communication between devices is feasible in the finally chosen configuration. It is worth noting that a full description of the received levels in terms of desired transmit signals and potential interferers (which can be intra-system, inter-system or background interference) is obtained and computed. The use of 3D Ray Launching is an adequate tradeoff between computational complexity and precision, leading to error minimization and hence to an optimal network configuration in a lower time frame within the design process.

**Figure 11.**
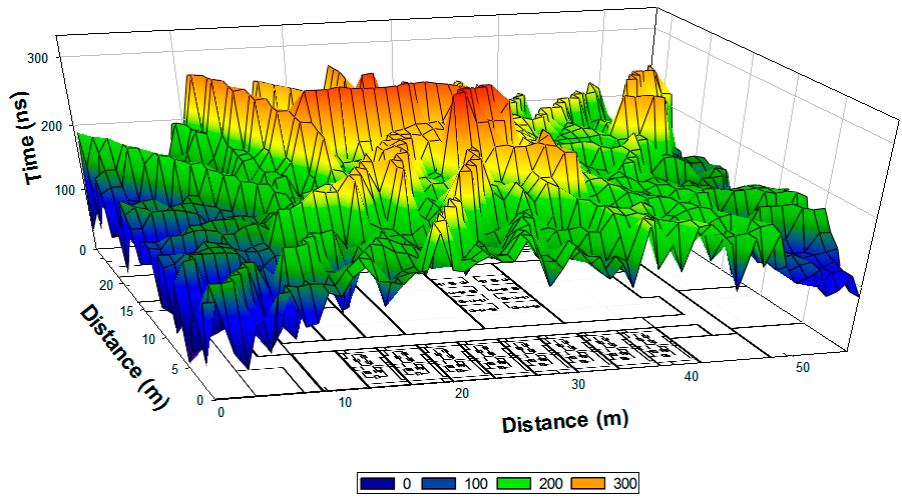
Delay spread when antenna 3 is transmitting for the plane at 1 m height.

As it is previously mentioned, the main propagation mechanism present in an indoor scenario with topological complexity is the multipath propagation, due to the strong presence of reflections, diffractions and refractions of the transmitted RF signals. The relevance of multipath propagation can be determined by estimating the values of all reflected components within the coherence bandwidth, given by the power delay profile and the delay spread. In Figure 11 the delay spread when antenna 3 is emitting is calculated, for a cut plane located at a height of 1 m. A larger time span can be seen in the center of the figure, due to the location of the antenna and consequently where higher power is observed. Therefore, the ricochets caused by the walls next to this point are more powerful and there are more probabilities for them to cross central points.

**Figure 12.**
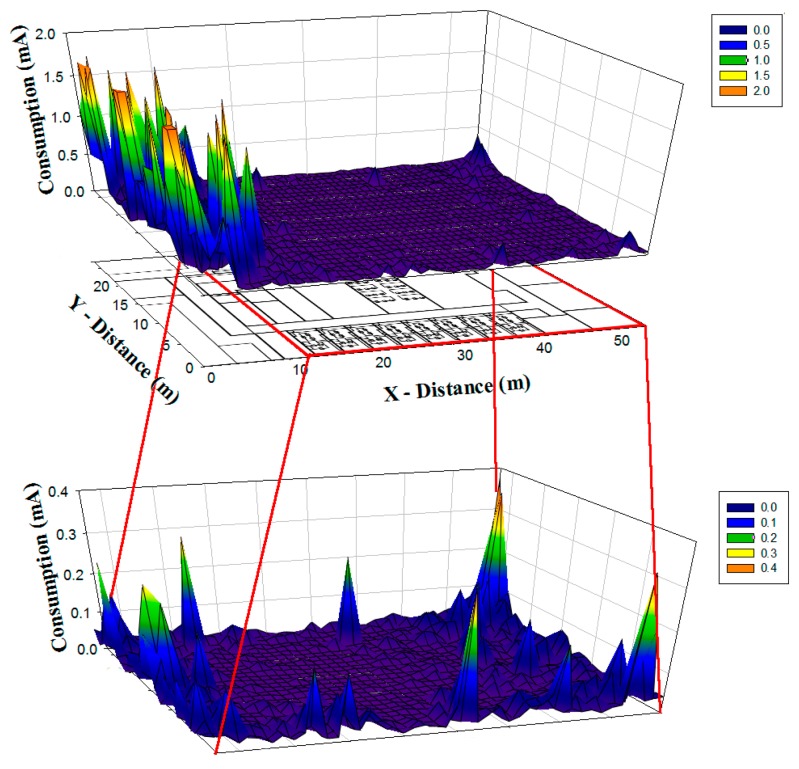
Estimation of Energy consumption for the antenna 3 in terms of current values in mA for different receiver locations within the scenario at height 1 m.

Since the system under analysis is based on ZigBee technology, the consumption of the deployed motes becomes essential in radio planning strategies. As seen previously, the location of the mote within the scenario has a significant impact in terms of the received power, which itself has a great impact on the power consumption of the transmitting devices, as the link balance varies. Based on the simulation results obtained by means of the 3D ray launching method, it is possible to estimate the increase of current consumption of the transmitter as a function of the receiver location. In order to obtain the current estimation, a specific calculation module within the 3D Ray Launching algorithm has been implemented, considering conventional transceiver current consumption values as a function of their modes of operation, enabling the possibility of depicting current consumption graphs within the simulation scenario. Figure 12 shows the current consumption increase map for the case of antenna 3 transmitting. The map represents the increase of current consumption of the transmitting device placed at antenna 3 point, for each possible receiver location within the scenario. As can be seen in Figure 12, the estimated energy consumption increase varies from 0.018 mA to 1.78 mA, depending on the placement of the receiver mote. As expected, if a receiver is placed at the left zone of the scenario, the consumption for the transmitter at antenna 3 point will has a great increase comparing to the rest of the scenario, due to the low power levels received in that area because the existence of a plumb wall.

**Figure 13.**
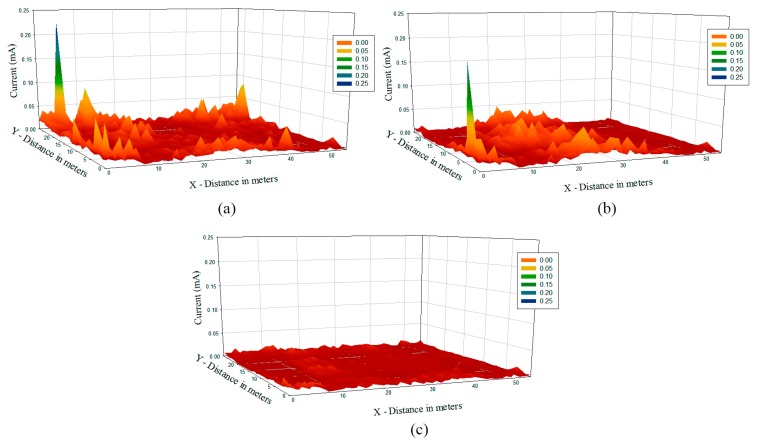
Current consumption in all over the scenario for the cases of three (**a**); four (**b**) and eight (**c**) emitters.

Finally, current consumption maps in all over the scenario for previously presented three different configurations are shown in Figure 13. As expected the energy consumption when eight antennas are emitting is considerably lower than in other cases and the difference in consumption level is also noticeable when three or four antennas are utilized.

Therefore, the optimum situation of the antennas is based on three premises, a constant distribution of power distribution all over the scenario, an appropriate SNR level and a low consumption. The reception of a high level of power in all points of the scenario provides not only a good user experience, but also decreases the current consumption of the devices and therefore the introduction of as many devices as possible may seem like a good idea. However, a higher number of antennas implies more inter-system interferences with the consequent degradation of SNR. Thus, a good balance between coverage and capacity, by means of deterministic system modelling (which considers network topology impact), is found with the aim of implementing an optimum communication system. The use of the 3D Ray Launching algorithm combined with the current estimation function aids in the optimal node location, which is achieved as a combination of the number of active transmitters, total number of users and external interference sources considered in the scenario. Due to the large variability in the possible combinations of the previous factors, the design procedure is site-specific, although as a general comment, the initial estimation of current consumption plots provides the initial location of the transceivers to be deployed, taking also into account capacity requirements, which will provide the total amount of required nodes.

## 3. Experimental Results

**Figure 14.**
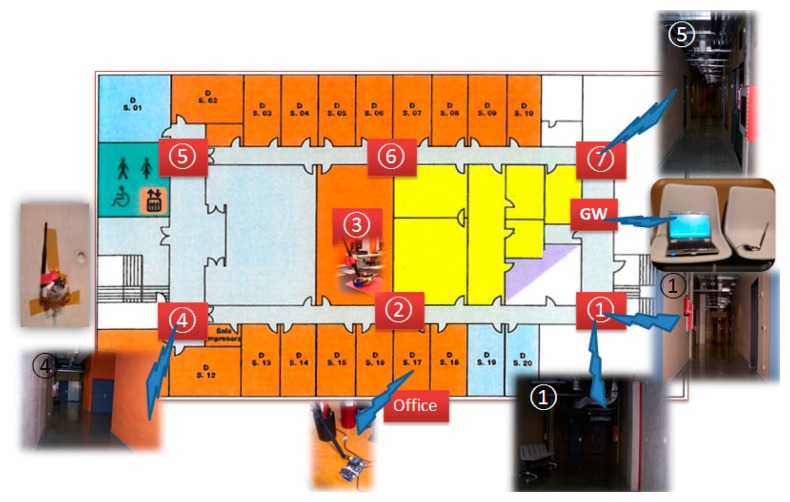
Map of the experimental scenario.

**Figure 15.**
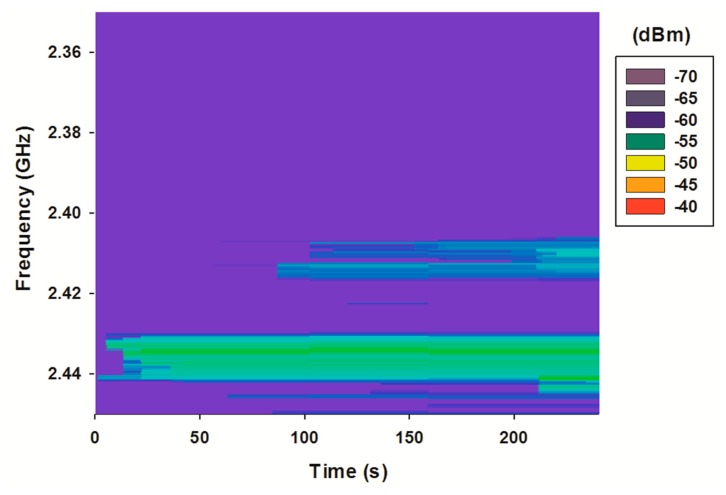
Measured spectrogram without operating WSN nodes.

Once the scenario has been characterized in terms of the topological dependence of the wireless channel performance, experimental results are obtained in order to validate these previous estimations. We have considered the basement of the “Las Encinas” building sited at the Arrosadia campus of the UPNA. This building is made of concrete and aluminum metalwork with double glazing. Offices (D01 to D020) are walled using plasterboard modules and aluminum metalwork. The inner walls of the central rooms are concrete, while doors are chipboard. Figure 14 depicts the experimental scenario where the WSN is deployed. We consider eight WSN nodes, where seven of them act as aggregators and the last one as gateway in charge of data collection. Those nodes communicate among them using the IEEE 802.15.4 standard. Due to the complexity of the environment, where several wireless signals could be propagating within it (e.g., WiFi), a spectrogram has been measured in order to gain knowledge about the radiofrequency pollution within the scenario, leading to an adequate and interference free wireless channel choice. Figure 15 shows the measured spectrogram for the scenario under analysis.

**Figure 16.**
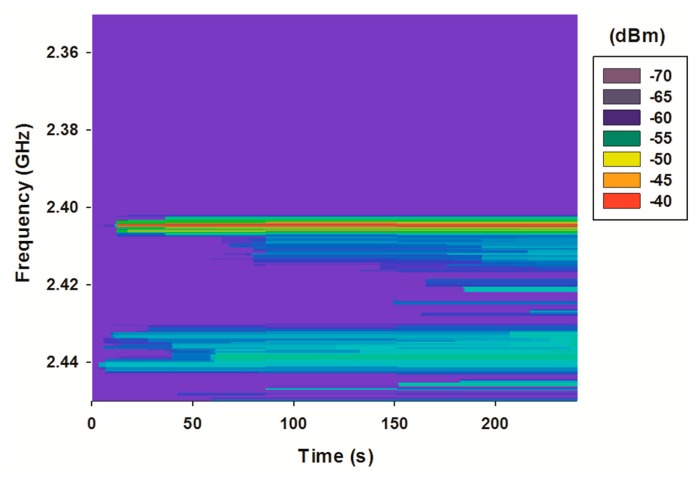
Measured spectrogram with the operating WSN network.

As can be seen, the ISM 2.4 GHz band, which is the frequency band used by the Waspmotes, contains a variety of external signals. Trying to avoid those interferences, the IEEE 802.15.4 channel 11 (2.400–2.405 GHz) has been chosen. In Figure 16 a new spectrogram is depicted, this time with the operating Wireless Sensor Network (WSN) nodes. The spectrum of the emitting nodes can be clearly seen, with a noticeable higher power level than the interferences. In fact, the SNR level produced by inter-system interferences in transmitter 3 (Figure 9) get values far away from the critical limit when the system is working in its highest bit rate.

Nodes have been placed next to the concrete walls at locations 1, 4, 5 and 7 (see Figure 8), next to plasterboard at locations 2 and 6, over a PC computer at location 3, over a plastic chair at location GW, and over a wooden table at location Office.

The wireless sensor network is built by following a chain where each node receives a message from its predecessor, aggregates its information, and sends a message to its successor without any acknowledgement or retransmission. That ensures a minimal message exchange and avoids collisions. Limitations may concern latency and packet fragmentation due to large payloads caused by data aggregation, but the reduced number of nodes (eight) involved in this WSN grants no packet fragmentation and an invaluable latency. Nodes are deployed following a chain where node #1 is the initiator and sends a message to its successor (#2) each 100 milliseconds. As node #1 does not receive any confirmation message, and no retransmission occurs, it does not experience any loss of messages. Each node aggregates three values to the message received: its node ID, the last RSSI value obtained and its battery voltage; and resends the message to its successor. At the end of the chain, node gateway (GW) collects all the information and stores it. The gateway is connected to a laptop through a USB cable. Information collected is processed and analyzed obtaining the results described below. Messages are exchanged between nodes without acknowledging, since information is sent frequently (100 milliseconds period) and acknowledgement messages introduce a high consumption with a null benefit.

**Figure 17.**
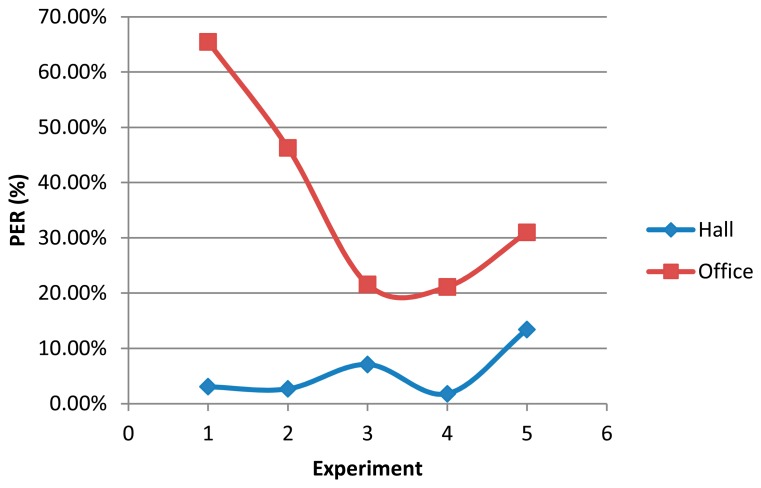
Packet error rate (PER) comparison between the hall and office base stations.

Fifty thousand (50,000) iterations distributed in five experiments of 10,000 iterations have been performed and the messages at two different locations have been collected: GW and Office (see Figure 14). Node #7 communicates directly (straight line) with node gateway, while it communicates by multipath with the office. Since node #7 and the data collector located in the office do not have straight line communication, but several thick concrete walls, the only effective mode of communication is multipath propagation. This implies a high rate of losses as depicted in Figure 17. Experiments 1 to 4 are performed with empty corridors, without people. The fifth experiment has been conducted with the presence of several people moving freely through the halls without any movement pattern. The higher packet error rate (PER) values obtained at the base station located on the office are due to the fact that office’s door was closed during measurements while in the other three experiments the door remained open.

**Figure 18.**
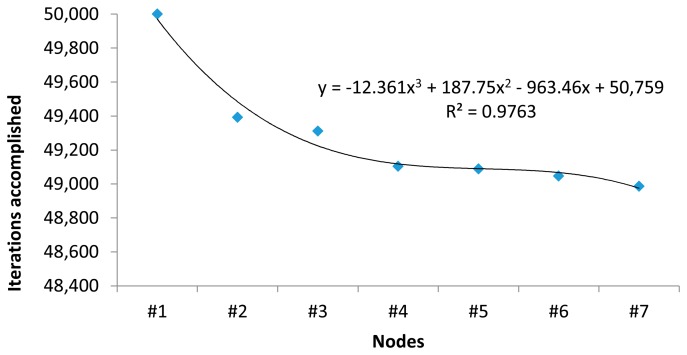
Iterations accomplished by nodes.

Figure 18 shows the number of iterations successfully accomplished by nodes. We obtain a PER value of 5.612%, and we can observe that node #2 acts as a traffic stopper and then, the rest of nodes have similar PER values. A greater stopping effect and a higher PER value are observed as the initiator’s transmission frequency increases.

**Figure 19.**
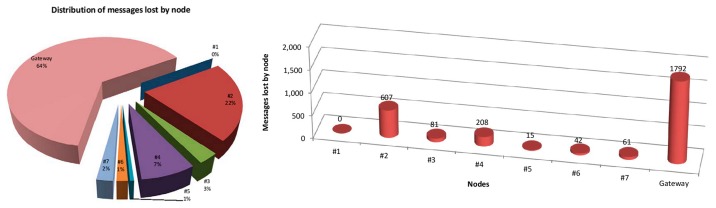
Distribution of messages lost by node.

Table 2 summarizes the packet error rate of the WSN. We can observe a very small PER, lower than 6%. Given its condition of chain initiator, node #1 does not receive any message and hence it’s PER is zero. The rest of nodes have a non-zero PER, where higher values correspond to nodes #2, #4 and gateway. Node #2 acts as a filter/stopper, ensuring a transmission rate that can be absorbed by the other nodes. Node #4, due to its low SNR, suffers a greater loss of packets, but it is not too relevant. Finally, the gateway has three times the PER of the node #2 mainly due the saturation of the receiver’s buffer (and then the loss of some messages) caused by multipath propagation. While multipath propagation makes possible the reception at the office location of messages sent by node #7, multipath propagation also causes a slight increase of the PER of the gateway located at the hall. However, the total amount of messages lost (2806) is considerably low with respect to the total number of cycles initiated (50,000). Figure 19 shows the distribution of messages lost by node (left), and also the number of messages lost by node.

**Figure 20.**
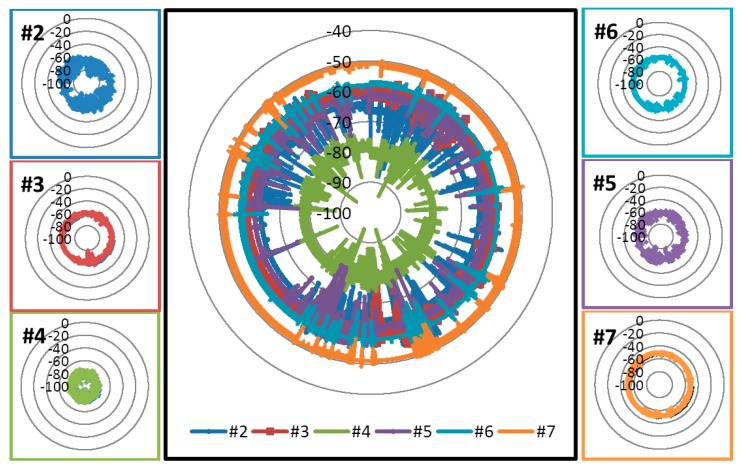
RSSI values (measured in dB) for each node.

The analysis of the RSSI values obtained for the nodes of the network shows a great difference between nodes #4 and #7, while the difference is not so great when considering the rest of nodes (#2, #3, #5 and #6). Figure 20 illustrates the RSSI obtained for each node and for the whole system. RSSI values for nodes #3, #6 and #7 are closer among them than those corresponding to nodes #2, #4 and #5.

The higher RSSI average value corresponds to node #6 (−57.838 dB), while the lower average value corresponds to node #4 (−79.275 dB). Standard deviation of the RSSI values range from 0.605 dB for the node #7 to 2.150 dB for the node #2. Mode and median values of RSSI match on all nodes, showing the considerable stability of the system, which is very important for the implementation of applications over the network. The maximum variation between maximum and minimum RSSI values corresponds to node #2 with 36 dB, while the minimum variation corresponds to node #7 with 11 dB. Table 3 summarizes these results.

**Figure 21.**
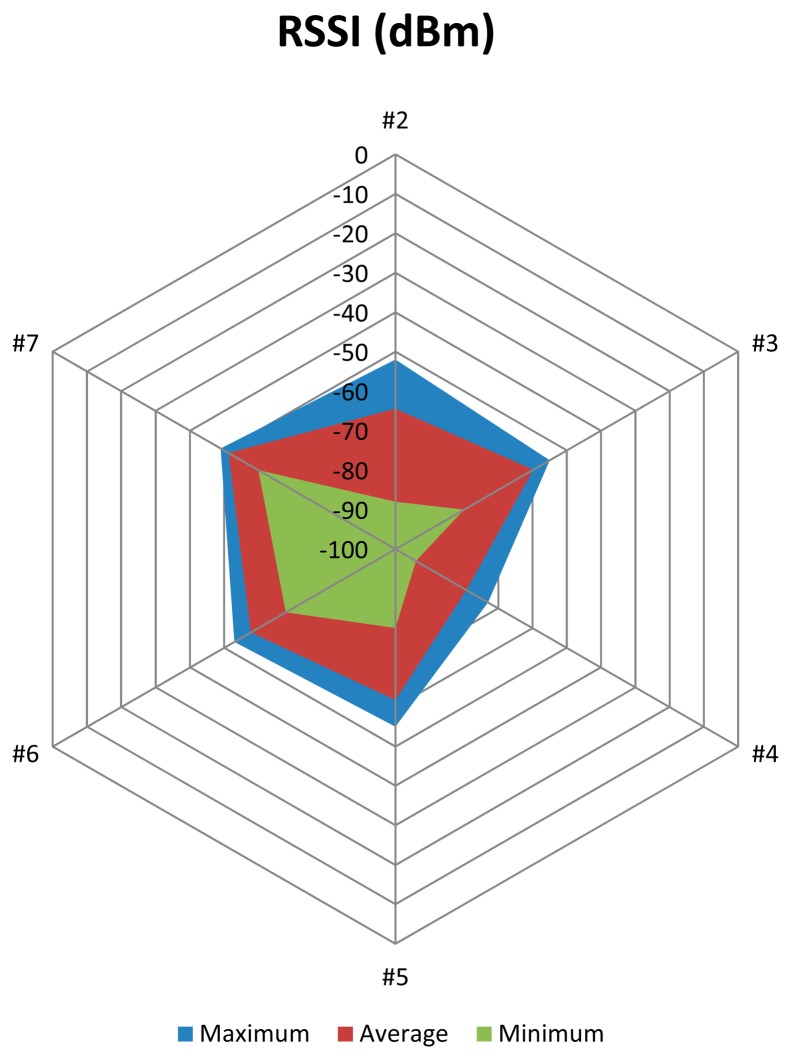
RSSI diagram.

The RSSI diagram depicted in Figure 21 shows that node #4 is the most conflictive of the network according to its RSSI values. However, the average values are far from the sensitivity threshold of the device (−95 dB) and node #4 has a reduced PER (0.42%). Mode and median values are −79 dB, which is sufficient to ensure the proper operation of the network.

**Figure 22.**
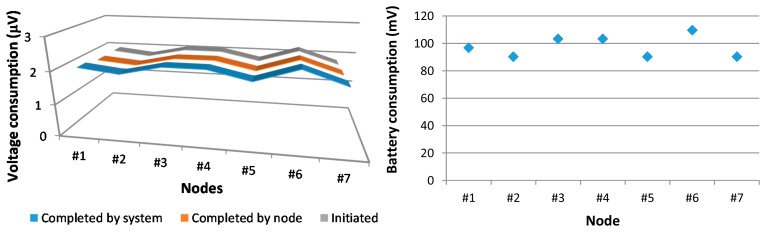
Battery consumption according to the number of iterations initiated, completed by node and completed by the system (**left**), and battery consumption (in millivolts) after 50,000 iterations initiated (**right**).

**Figure 23.**
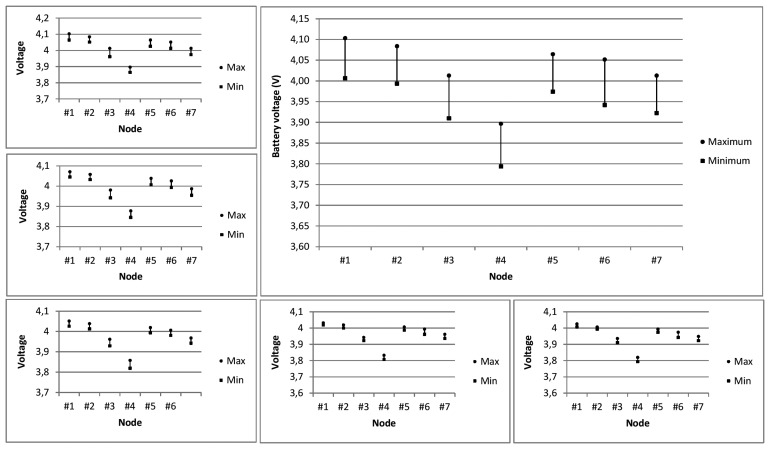
Battery voltage by node and experiment, and final battery voltage after 50,000 iterations (large figure).

Once radio signal level as well as quality (in terms of signal to noise as well as Packet Error Ratio levels) has been obtained, the battery consumption of the WSN will be analyzed. For such purpose we distinguish between the number of iterations initiated by the network, the number of iterations correctly completed by each node and the number of iterations successfully completed by the network. Figure 22 (left) depicts the average battery consumption in microvolts according to such criteria. Figure 22 (right) shows the battery consumption (in millivolts) after 50,000 iterations. Note that nodes with a note that the nodes with fewer losses are those which have a greater number of transmissions. Although by the definition of the communication protocol node #1 does not receive messages from any of its neighbors, in fact, its radio interface receives messages and therefore, energy consumption occurs. The battery consumption of the nodes after 50,000 iterations is close to 100 mV. Also note that all nodes do not perform the same number of transmissions, since, as they advance along the chain, fewer messages are transmitted. Although it must be remembered that only 2806 messages are lost. Figure 23 depicts, in more depth, the battery consumption of each node.

In order to validate the estimations given by the radio channel 3D Ray Launching code, simulation and measurements results have been correlated comparing RSSI values with 3D Ray Launching results previously obtained, as shown in Table 4. In this case the value has been obtained in the radio link established from the preceding WSN nodes, e.g., when first was emitting, the RSSI value received in the second node was obtained and when second was emitting the value was acquired from third, *etc.*

A mean error of 1.4 dB has been obtained in this comparison, validating the use of the 3D Ray Launching tool in order to adequately locate the proposed nodes of a WSN under design phases.

## 4. Conclusions

In this paper, deterministic radioplanning techniques have been applied in order to fully determine the characteristics of the wireless channel for each one of the nodes of a Wireless Sensor Network in an indoor scenario. By taking into account all of the elements of the scenario, such as walls, hallways and furniture, detailed information on the topological dependence of the network layout can be obtained. This influences not only the expected values of coverage of each individual node, but also determines maximum capacity performance, due to the fact that interference levels are also dependent on the network topology. Estimations have been obtained with the aid of deterministic 3D Ray Launching code implemented in-house at Public University of Navarre and validated with a real WSN deployed in a real scenario. Parameters such as RSSI, Signal to Noise ratio and Packet Error Ratio have been compared numerically and with measurement results, showing good agreement. The consideration of factors such as topology and interference levels (given by multiple elements, such as variable user number and location) provide information which can be directly related to parameters such as coverage, capacity or energy consumption. Moreover, the results obtained by the use of 3D deterministic tools provide assessment in network planning design of confined scenarios, in which initial considerations may lead to the increase in required transmission nodes or the modification of the final locations of the full set of deployed nodes, as has been shown in this work.

In comparison with commonplace approaches for wireless system and wireless sensor network deployment design and analysis, the use of 3D Ray Launching technique provides precise information for complex scenarios (in terms of size and number of sensors/transceivers/users), with the capability of considering site specific characteristics without the need of extensive measurement campaigns. Given the estimations in the growth of wireless sensor deployments, mainly due to the implementation of Context Aware environments, the proposed method provides a novel approach to provide initial location of nodes as well as an estimation of the total amount of nodes required, as a function of coverage limitations and capacity requirements.

The use of deterministic planning tools in the design phases of WSN in complex indoor scenarios can lead to cost efficient network designs, in which interference levels and energy consumption levels can be minimized, whilst maximizing overall WSN capacity.

## Figures and Tables

**Table 1.** Considered parameters for the Ray Launching simulation.

**Parameter**	**Value**
Frequency	2.405 GHz
Transmitted power	0 dBm
Antenna gain	5 dBi
Horizontal plane angle resolution (ΔΦ)	1°
Vertical plane angle resolution (Δθ)	1°
Maximum permitted reflections	5
Cuboids resolution	1 m × 1 m × 1 m

**Table 2.** Packet error rate (PER) by node.

**Messages Lost by Node**	**#1**	**#2**	**#3**	**#4**	**#5**	**#6**	**#7**	**Gateway**
**Average**	0.00%	1.21%	0.16%	0.42%	0.03%	0.08%	0.12%	3.58%

**Table 3.** RSSI statistics for the indoor scenario.

**Statistical Operator**	**Node**					

	**#2**	**#3**	**#4**	**#5**	**#6**	**#7**
Average	−64.4467	−59.7705	−79.2746	−61.7483	−57.8385	−51.3382
Std. Deviation	2.1496	0.8942	1.5119	1.3928	0.7642	0.6049
Maximum	−52	−55	−73	−55	−53	−49
Minimum	−88	−80	−94	−80	−68	−60
Mode	−64	−60	−79	−62	−58	−51
Median	−64	−60	−79	−62	−58	−51

**Table 4.** Comparison between RSSI and simulation power values.

**Measurement Results (dBm)**	**Simulation Results (dBm)**
−6562	−6586
−6211	−5852
−7913	−7490
−6674	−6606
−5711	−5640
−6331	−6212
−5799	−5706
